# The International Trifecta^TM^ and Epic^TM^ Valve‐in‐Valve Registry: Insights Into Clinical & Hemodynamic Outcomes

**DOI:** 10.1002/ccd.31492

**Published:** 2025-03-27

**Authors:** Matthias Raschpichler, Mohamed Abdel‐Wahab, Nick Curzen, Manuel Wilbring, Christoph Dubois, Kayan Lam, Gloria Faerber, Jana Nagel, Holger Thiele, Michael A. Borger

**Affiliations:** ^1^ Leipzig Heart Center University Clinic of Cardiac Surgery Leipzig Germany; ^2^ Department of Internal Medicine/Cardiology Leipzig Heart Center Leipzig Germany; ^3^ Wessex Cardiothoracic Centre University Hospital Southampton Southampton UK; ^4^ Heart Center Dresden University Hospital Dresden Dresden Germany; ^5^ University Hospital Leuven Leuven Belgium; ^6^ Heartcentre Catharina Hospital Eindhoven Netherlands; ^7^ University Clinic of Cardiothoracic Surgery Jena Germany; ^8^ Helios Health Institute, Heart Center Leipzig Leipzig Germany

**Keywords:** coronary obstruction, Epic^TM^‐valve, externally mounted leaflets, Trifecta^TM^‐valve, valve‐in‐valve transcatheter aortic valve replacement

## Abstract

**Background:**

Little is known about the clinical and hemodynamic outcome of valve‐in‐valve transcatheter aortic valve replacement (ViV‐TAVR) for failed Trifecta surgical aortic bioprotheses.

**Aims:**

We aimed to compare outcomes of valve‐in‐valve transcatheter aortic valve replacement (ViV‐TAVR into failed Trifecta^TM^ vs. ViV‐TAVR into a standard aortic bioprosthetic valve with internally mounted leaflets (Epic^TM^, Abbott, Minneapolis, MN).

**Methods:**

Data of consecutive patients who underwent ViV‐TAVR into either failed Trifecta^TM^ or Epic^TM^ bioprostheses between October 2015 and June 2020 were retrospectively collected within the International Trifecta and Epic Valve‐in‐Valve Registry, and analyzed for a primary composite outcome of 30‐day mortality and/or coronary obstruction (CO), defined as: (1) CO resulting in myocardial infarction and/or cardiogenic shock, or (2) CO requiring emergent coronary intervention.

**Results:**

A total of 76 patients (49 Trifecta, 27 Epic) with a median age of 80 years (interquartile range [IQR] 75.0; 82.0]) and a median Society of Thoracic Surgeons‐score of 5.4 (IQR 4.0; 9.8) were identified. Coronary protection techniques were more frequently performed in Trifecta than Epic patients (29.6% vs. 0%, *p* = 0.01). The primary composite outcome was observed in three Trifecta versus five Epic cases (6.1% vs. 20%, *p* = 0.1), which included one case of CO following ViV‐TAVR into Epic requiring stenting. Increased rates of patient‐prosthesis mismatch (PPM) following valve‐in‐Epic were found (41.7% vs. 75%, *p* = 0.08). Survival at a median of 365 days was 86.2% and did not differ between groups (log‐rank *p* = 0.37).

**Conclusions:**

Compared to a stented prosthesis without increased risk of CO, ViV‐TAVR into Trifecta prostheses can be performed with low risk of CO and acceptable short‐term clinical outcomes. As the rate of post‐ViV PPM is substantial for both prostheses, careful patient selection is warranted. (NCT05389631).

## 1 Introduction

Valve‐in‐Valve transcatheter aortic valve replacement (ViV‐TAVR) has become the treatment of choice for many patients presenting with deteriorated surgical aortic bioprostheses at higher surgical risk [1, 2]. Most observational studies have demonstrated superior short‐term clinical outcome of ViV‐TAVR compared to redo surgical aortic valve replacement (rSAVR), while the benefits regarding long‐term outcome remain unclear [3, 4, 5, 6]. Downsides of ViV‐TAVR include inferior hemodynamic performance compared to rSAVR, increased risk of thrombogenicity of ViV‐TAVR prostheses, and greater difficulty to access coronary arteries postprocedure [7, 8, 9]. Although rare, periprocedural coronary obstruction (CO) is a feared, and often lethal, complication. Risk factors associated with CO following ViV‐TAVR include narrow sinotubular dimensions relative to the ViV‐TAVR prostheses and stentless surgical prostheses or those with externally mounted leaflets [10].

In patients at risk of CO, preventive measures such as coronary wiring, pre‐emptive stenting, or bioprosthesis leaflet laceration (e.g., the BASILICA procedure), can reduce both occurrence and consequences of CO [11, 12]. These techniques are particularly relevant for stented bioprostheses with externally mounted leaflets designed to provide the largest possible opening area, such as the Mitroflow (LivaNova, London, UK) or the Trifecta (Abbott, Minneapolis, MN) prostheses. The Trifecta valve is a tri‐leaflet supra‐annular bovine pericardial prosthesis with leaflets mounted on the outside of a polyester‐covered titanium stent to maximize valve opening [13]. The titanium stent cannot be fractured with a valvuloplasty balloon, but it is possible to deform the stent posts outwardly [14, 15]. Unfortunately, several reports have demonstrated limited durability compared to other stented prostheses [16, 17, 18, 19, 20]. Although many patients require re‐intervention when rSAVR is considered high risk, data on ViV‐TAVR for failed Trifecta prostheses remains limited. In comparison, the Epic Supra valve (Abbott, Minneapolis, USA) is a supra‐annular valve from the same manufacturer but designed with leaflets mounted internally. It therefore allows the comparison to a potentially problematic valve.

The current study compares the clinical and hemodynamic outcome of patients undergoing ViV‐TAVR for deteriorated Trifecta and Epic^T^ aortic bioprostheses from a multicenter European registry.

## 2 Methods

### 2.1 Data Availability Statement

Data and statistical analysis underlying this article can be shared upon reasonable request.

### 2.2 Study Population

We retrospectively collected baseline, procedural, hemodynamic, and clinical data of consecutive patients that underwent ViV‐TAVR for deteriorated Trifecta or Epic aortic bioprostheses at six European centers between October 2015 and June 2020 within The International Trifecta and Epic Valve‐in‐Valve Registry (*NCT05389631*). Exclusion criteria included (1) ViV‐TAVR of an aortic valve bioprosthesis other than the Trifecta or Epic valve, and (2) patients undergoing combined multiple transcatheter valve procedures in addition to ViV‐TAVR. This retrospective study was approved by the ethics committees of each participating center (Supporting Information S1: Table 1). Funding was provided by Abbott Structural Heart.

The Trifecta GT valve is a second‐generation valve that was introduced in 2016 and has additional features intended to make the valve easier to implant. The Epic Supra valve (Abbott, Minneapolis, USA) is a supra‐annular triple composite porcine valve with leaflets mounted internally onto a low profile FlexFit polyester‐covered polymer stent [21]. The stent frame can be fractured with a valvuloplasty balloon at 8 atmospheres [14].

### 2.3 Outcomes of Interest

The primary outcome of interest was a composite of 30‐day all‐cause mortality and/or clinically significant CO defined as: (1) CO resulting in myocardial infarction and/or cardiogenic shock, or (2) CO requiring emergent coronary intervention. Secondary outcomes of interest included selected Valve Academic Research Consortium (VARC)3‐based outcomes including cardiac structural complications and early safety [22]. All variables were collected locally. Median follow‐up was 365 days.

### 2.4 Statistical Analysis

Normality of data was tested using Shapiro‐Wilk test. Continuous variables of normal distribution were compared using student *t*‐test and are shown as mean ±standard deviation (SD). Continuous variables without normal distribution were compared using Wilcoxon rank‐sum or Kruskall‐Wallis test and are shown as median ± interquartile range (IQR). Categorical variables were compared using Chi^2^ and are shown as frequencies and percentages. Missing variables were tested both numerically and graphically for patterns of missingness and subsequently treated as missing at random. Using the difference of the surgical prosthesis as well as baseline and CT‐based parameters that differed at a level of *p* < 0.1 as independent variables, and the primary composite outcome as the dependent variable within uni‐variable binomial regression, we tested all variables with a p‐value of less than 0.1 as well as clinically relevant variables using the Wald‐test as well as Pseudo‐R^2^ measures as criteria within multivariable analysis. Midterm clinical outcome was analyzed using the Kaplan‐Meier curve and the log‐rank test. Statistical analysis was performed using base R functions (R version 4.2.1) within RStudio (version 1.4.1103), as well as the following R packages: *tidyverse*, *compareGroups*, *ggthemes*, *ggsci*, *ggpubr*, *aod*, *pscl*, *janitor*, *reshape2*, *survival*, and *survminer*. The manuscript was written using *R Markdown* and *Knitr* in adherence to principles of reproducible research.

## 3 Results

### 3.1 Study Population & Baseline Characteristics

Seventy‐six patients (median age 80 years, interquartile range [IQR] 75; 82]; 46.1% females) with a median Society of Thoracic Surgeons‐score of 5.4 [4; 9.8] were identified (Table 1). The 76 implanted surgical prostheses included Epic standard valve (*N* = 23), Epic Supra valve (*N* = 4), Trifecta valve (*N* = 47), and Trifecta GT valve (*N* = 2). Trifecta patients were more frequently female (63.3% vs. 14.8%, *p* < 0.001) and had lower rates of previous myocardial infarction (2.0% vs. 14.8%, *p* = 0.05). All Epic prostheses showed relevant stenosis (63.8% vs. 100% for Trifecta vs. Epic, respectively, *p* = 0.003), while approximately 2/3 of Trifecta valves had relevant prosthesis regurgitation (66.7% vs. 22.7%, *p* = 0.002), with greater valve effective orifice areas compared to Epic prostheses (0.95 vs. 0.80 cm^2^, *p* = 0.06). Small surgical valves (i.e., labeled valve diameter < 23 mm) were more frequent in the Trifecta group (42.9% vs. 11.1%, *p* = 0.01).

**Table 1:** Baseline characteristics.

	Epic (*N* = 27)	Trifecta (*N* = 49)	*p* value
Age (years)	80.0 [78.0; 84.5]	79.0 [75.0; 82.0]	0.184
Female	4 (14.8%)	31 (63.3%)	< 0.001
BSA (m^2^)	1.87 [1.78; 1.95]	1.82 [1.71; 1.91]	0.385
STS PROM Score	7.73 [4.12; 10.3]	5.00 [4.02; 8.53]	0.300
NYHA > I	25 (92.6%)	46 (97.9%)	0.550
Prior Stroke	2 (7.41%)	3 (6.12%)	1.000
Prior MI	4 (14.8%)	1 (2.04%)	0.051
Arterial Hypertension	23 (85.2%)	45 (91.8%)	0.444
Insulin‐Dependent Diabetes Mellitus	3 (11.1%)	10 (20.4%)	0.359
Chronic Renal Failure	9 (33.3%)	18 (36.7%)	0.963
PCI Within 3 Months Prior	2 (7.41%)	6 (13.3%)	0.701
Prior CABG	13 (48.1%)	17 (34.7%)	0.366
Permanent Pacemaker	5 (18.5%)	13 (26.5%)	0.614
LVEF (%)	55.5 [42.8; 65.0]	57.6 [44.2; 62.2]	0.794
Aortic Stenosis > mild	21 (100%)	28 (68.3%)	0.003
EOA	0.80 [0.55; 0.90]	0.95 [0.62; 1.30]	0.055
AV Mean Pressure Gradient	36.0 [30.0; 45.0]	30.5 [18.6; 45.2]	0.129
AV Peak Pressure Gradient	65.0 [51.2; 77.8]	54.9 [32.5; 77.5]	0.161
Aortic Regurgitation > mild	5 (22.7%)	30 (66.7%)	0.002
Mode of Failure: Predominant AS	17 (77.3%)	15 (33.3%)	0.002
Mode of Failure: Predominant AR	0 (0.00%)	13 (31.7%)	0.003
Mode of Failure: Mixed	3 (15.0%)	12 (30.0%)	0.343

*Note:* Values are expressed as total numbers (percentage) and median (Interquartile Range).

Abbreviations: AR, Aortic Regurgitation; AS, Aortic Stenosis; AV, Aortic Valve; BSA, Body Surface Area; CABG, Coronary Artery Bypass Grafting; EOA, Effective Orifice Area; LVEF, Left Ventricular Ejection Fraction; MI, Myocardial Infarction; NYHA, New York Heart Association; PCI, Percutaneous Coronary Intervention; SAVR, surgical aortic valve replacement; STS PROM, Society of Thoracic Surgeons Predicted Risk of Mortality; TIA, Transitory Ischemic Attack.

### 3.2 Computed Tomography Measures & Procedural Characteristics

Median time between surgery and ViV‐TAVR was 6.4 years (4.5; 8.0; Table 2). Selected CT‐based and procedural characteristics of the two ViV sizes predominantly utilized (i.e., 23 mm and 26 mm valve size) are shown in Supplemental Table 2. Access for ViV‐TAVR was transfemoral in all but one Trifecta case. Smaller aortic annulus and surgical prosthesis diameters were present in patients undergoing valve‐in‐Trifecta. Coronary protection was performed in eight Trifecta cases (29.6%; four leaflet laceration procedures; three protective wiring; one preventive stenting) versus 0 Epic cases (*p* = 0.006). Coronary obstruction occurred in one case following valve‐in‐Epic. The obstructed left main coronary artery was stented and the patient was discharged uneventfully. Supporting Information S1: Table 4 shows selected variables of cases of coronary protection or coronary obstruction.

**Table 2:** Computed tomography and procedural characteristics.

	Epic (*N* = 27)	Trifecta (*N* = 49)	*p* value
Time between Interventions (years)	5.39 [4.08; 10.4]	6.55 [4.65; 7.74]	0.978
Surgical Valve Diameter < 23 mm	3 (11.1%)	21 (42.9%)	0.010
Annulus Maximal Diameter (mm)	24.0 [22.2; 26.0]	22.0 [20.0; 24.1]	0.088
Annulus Mean Area (mm^2^)	383 [347; 415]	344 [285; 380]	0.046
Left Coronary Artery Height (mm)	10.0 [9.50; 12.0]	8.00 [6.00; 11.0]	0.113
Right Coronary Artery Height (mm)	13.0 [9.50; 16.5]	10.0 [6.95; 14.0]	0.071
SoV Width (mm)	33.0 [31.0; 36.5]	32.0 [30.0; 35.0]	0.291
STJ Width (mm)	29.8 [29.0; 32.0]	28.0 [26.0; 30.4]	0.250
Predilatation	12 (44.4%)	11 (22.9%)	0.093
Any Coronary Protection Maneuver	0 (0.00%)	8 (29.6%)	0.006
Leaflet Laceration (BASILICA)	0 (0.00%)	4 (14.3%)	0.117
Self‐expandable ViV Prosthesis	14 (51.9%)	30 (61.2%)	0.583
ViV Size:			0.074
20	1 (3.70%)	0 (0.00%)	
23	13 (48.1%)	35 (71.4%)	
26	12 (44.4%)	11 (22.4%)	
29	1 (3.70%)	3 (6.12%)	

*Note:* Values are expressed as total numbers (percentage) and median (Interquartile Range).

Abbreviations: SoV, Sinus of Valsalva; STJ, Sinutubular Junction; ViV, Valve‐in‐Valve.

### 3.3 Clinical and Hemodynamic Outcome

The primary composite outcome of all‐cause mortality or CO was observed in 8 cases, three times more frequently in patients undergoing valve‐in‐Epic (20.0% vs. 6.4%, *p* = 0.1, Table 3). Adverse clinical events included two cases of VARC3‐based cardiac structural or other/technical complications in Trifecta patients, and one Trifecta case of valve malpositioning, resulting in comparable rates of VARC3‐based early safety. Multivariable logistic regression including *prosthesis type* and *STS‐score* as independent variables revealed that prosthesis regurgitation was independently and inversely associated with the primary composite outcome (Supporting Information S1: Table 3).

**Table 3:** Clinical and hemodynamic outcome.

	Epic (*N* = 27)	Trifecta (*N* = 49)	*p* value
**In‐Hospital Outcome**			
Mortality	2 (8.00%)	3 (6.1%)	1.000
Coronary Obstruction	1 (3.70%)	0 (0.00%)	‐‐‐
Stroke	2 (7.41%)	2 (4.08%)	0.612
Minor Vascular Complication^a^	5 (18.5%)	7 (14.3%)	0.745
Early Safety^a^	18 (78.3%)	38 (90.5%)	0.260
Mean AV Gradient [mm Hg]	17.9 [12.0; 21.0]	15.9 [10.5; 20.0]	0.294
Peak AV Gradient [mm Hg]	25.5 [21.5; 34.5]	26.1 [18.1; 34.5]	0.488
AV Residual Gradient (≥20 mmHg)	10 (43.5%)	11 (25.6%)	0.226
iEOA	0.73 [0.60; 0.83]	0.81 [0.74; 1.01]	0.087
Moderate or Severe PPM	12 (75.0%)	10 (41.7%)	0.080
**Outcome at 30 Days**			
Primary Outcome (Mortality or CO)	5 (20.0%)	3 (6.1%)	0.116
LVEF [%]	57.0 [45.0; 65.0]	59.0 [55.0; 62.0]	0.530
Mean AV Gradient [mm Hg]	15.6 [11.2; 26.3]	12.8 [10.1; 17.8]	0.097
Peak AV Gradient [mm Hg]	32.0 [22.9; 41.8]	22.4 [15.8; 29.9]	0.057

*Note:* Values are expressed as total numbers (percentage) and median (Interquartile Range).

Abbreviations: AR, Aortic Regurgitation; AV, Aortic Valve; iEOA, Indexed Effective Orifice Area; PPM, Patient Prosthesis Mismatch.

a Based on Valve Academic Research Consortium‐3.

**Figure 1:**
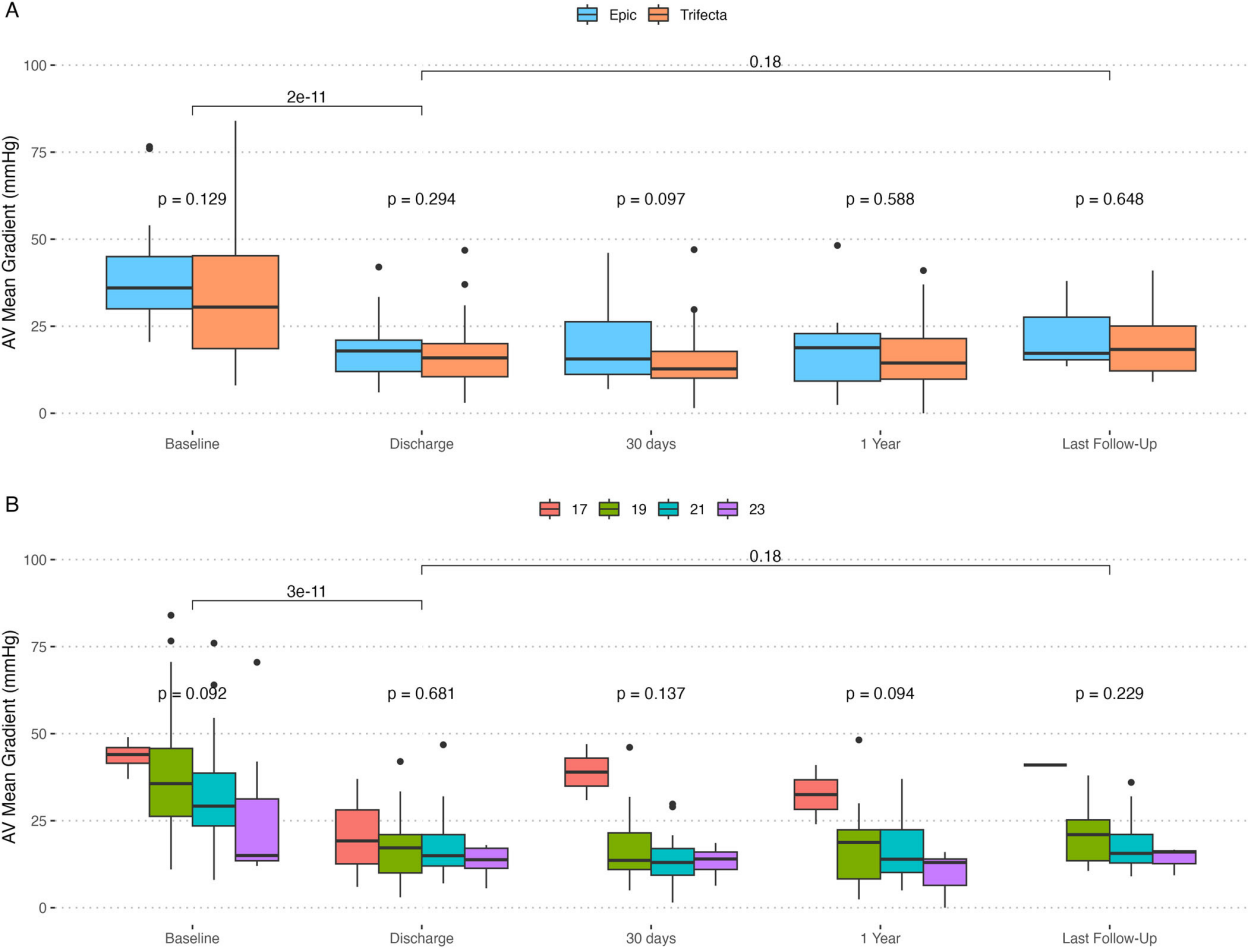
Graphs depicting mean valve gradient by (A) prosthesis type (blue = Epic, orange = Trifecta, and (B) true internal diameter of the surgical prosthesis. Gradients improved significantly by discharge and improvements were maintained until follow up. [Color figure can be viewed at wileyonlinelibrary.com]

**Figure 2:**
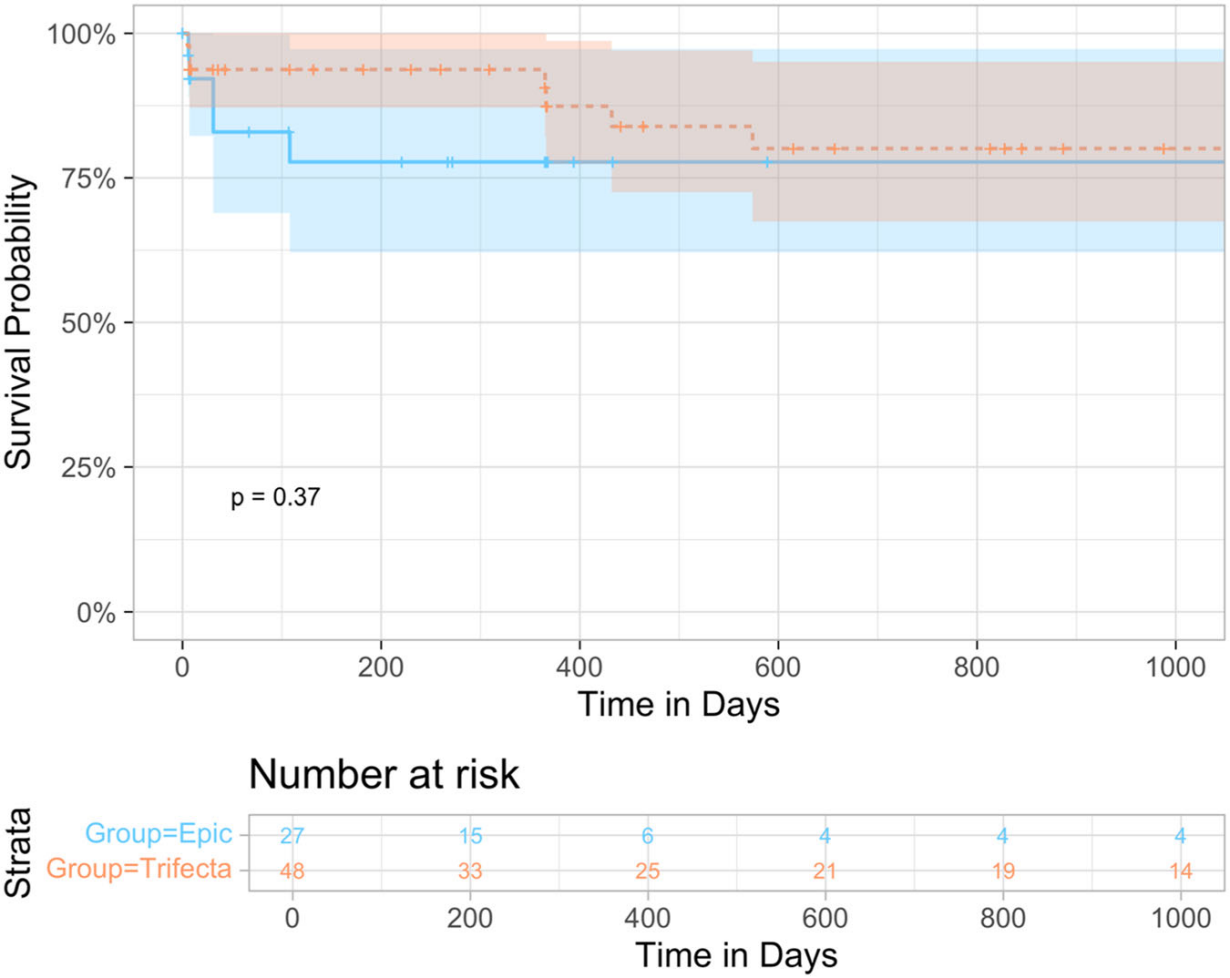
Curves depict Kaplan‐Meier time‐to‐event rates for all‐cause mortality by prosthesis type (blue = Epic, orange = Trifecta). [Color figure can be viewed at wileyonlinelibrary.com]

Hemodynamic performance improved significantly following ViV‐TAVR and remained improved through last follow‐up (Figure 1; Supporting Information S1: Table 5). Although overall comparable between groups, valve‐in‐Trifecta resulted in lower mean and peak gradients at 30 days (12.8 vs. 15.6 mmHg and 22.4 vs. 32 mmHg; *p* = 0.1 and 0.06, respectively); larger aortic valve area at both discharge and 1 year postprocedure (1.9 vs. 1.3 cm^2^ and 1.7 vs. 1.1 cm^2^, respectively, *p* = 0.02); and lower rates of PPM and thus VARC3‐based nonstructural valve dysfunction (41.7% vs. 75.0%, *p* = 0.08; Supporting Information S1: Figure 1). Survival at a median of 365 days did not differ between groups (log‐rank *p* = 0.37; Figure 2).

## 4 Discussion

The International Trifecta and Epic Valve‐in‐Valve Registry assessed the clinical and hemodynamic outcome of ViV‐TAVR, comparing surgical aortic bioprostheses with and without increased risk of CO based on the mounting of the leaflets. We found that: (1) ViV‐TAVR for deteriorated Trifecta‐valves was performed without major complications in more than 90% of the cases using measures to prevent CO, in 29.6% of the Trifecta patients; (2) hemodynamic outcome post‐Trifecta ViV‐TAVR is acceptable and appears to be comparable to other ViV‐cohorts; (3) midterm survival may be comparable to other failed prostheses treated in similar patient populations [2].

Although our cohort was comparable to most previous reports on ViV‐TAVR, some aspects are worth highlighting. First, the Trifecta patients represent a subgroup of individuals at increased risk for PPM post ViV‐TAVR due to small annular dimensions relative to body surface area (BSA). Approximately 2/3 of Trifecta patients were female, with annulus diameters unsuitable for prosthesis larger than 22 mm in diameter on average, while BSA did not differ between groups. At ViV‐TAVR, this did not result in lower baseline effective orifice areas (EOA) in Trifecta patients, likely due to the mode of failure which included prosthesis regurgitation in 2/3 of the cases. Nevertheless, small surgical valves (i.e., labeled valve diameter < 23 mm) were more frequent in the Trifecta group, accounting for 87.5% of all cases of small valves, of which in turn 83.3% were female. Conversely, 57.1% of all female patients received surgical prostheses smaller than 23 mm at the index SAVR procedure. Although the EOA and short‐term hemodynamic outcome may be superior in prostheses with externally compared to internally mounted leaflets, the titanium stent ring ultimately limits the size of the ViV‐TAVR prosthesis, since the Trifecta valve cannot be fractured [14, 15, 23]. This may result in PPM after ViV‐TAVR, which is known to be associated with worse long‐term clinical outcomes [24]. Reporting on the outcome of ViV‐TAVR for these prostheses, therefore, highlights the life‐time consequences of avoiding root enlargement techniques in small aortic roots at the initial SAVR procedure (Central Figure 1).

**Central Figure 1:**
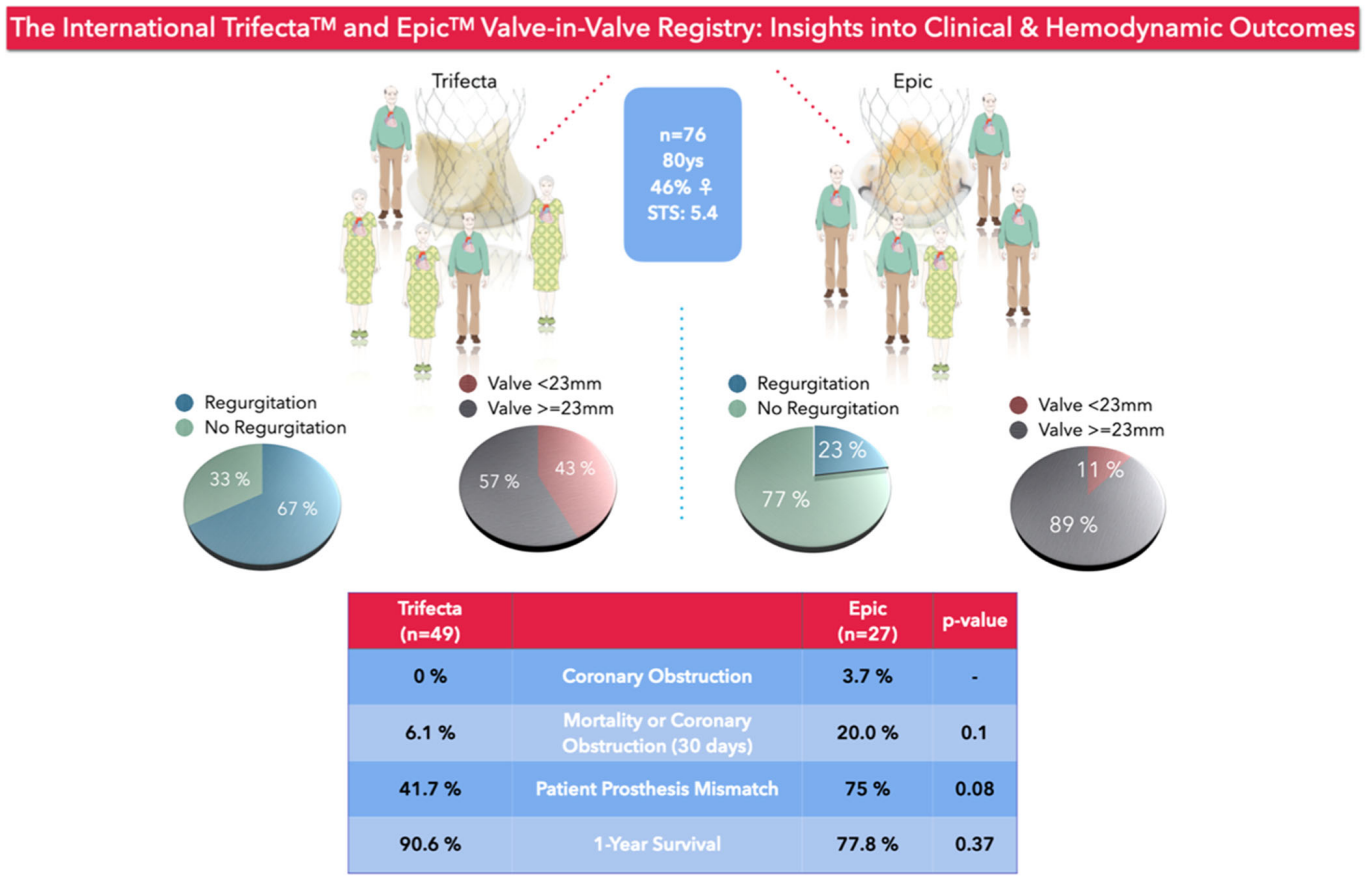
Figure illustrating the two groups undergoing ViV‐TAVR for failed Trifecta (left) and Epic (right column) valves. [Color figure can be viewed at wileyonlinelibrary.com]

Coronary obstruction is a rare, but often lethal, complication following ViV‐TAVR [10]. With a single case of CO in our series managed successfully with coronary stenting, our data confirm that CO is infrequent, even when including prostheses at higher risk of this complication. It should be noted, however, that preventive measures such as leaflet laceration or coronary wiring were utilized exclusively in Trifecta cases. We did not have access to the number of patients screened for a ViV‐TAVR and were therefore unable to assess how many patients with an indication for repeat intervention were indeed referred for intervention. Thus, the degree of selection bias cannot be evaluated, and it remains unclear whether our results can be generalized to other prostheses with externally mounted leaflets. Nevertheless, recent data on coronary protection and CO in 250 cases of aortic ViV‐TAVR including 28% with externally mounted leaflets demonstrated higher rates of CO or stent deployment compared to those with internally mounted leaflets, with no difference in mortality after 2 years [12]. Notably, coronary protection was performed in 79% of the cases with externally mounted leaflets (vs. 6% for other prostheses), indicating the importance of patient selection and pre‐procedure planning with a low threshold to utilize coronary protection [12, 25].

Finally, hemodynamic outcome in our data demonstrate that although valve‐in‐Trifecta may be technically challenging, it can also lead to better valve opening area compared to prostheses without externally mounted leaflets. At least moderate PPM was found more frequently following valve‐in‐Epic, resulting in 75% of patients meeting the VARC3‐based endpoint of stage‐1 nonstructural valve dysfunction [22]. This is an interesting finding, because neither choice of TAVR prosthesis (balloon‐ vs. self‐expandable) nor procedural characteristics such as pre‐ or post‐dilatation and bioprosthetic valve fracture differed between groups. In fact, the latter were used numerically less frequently in Trifecta patients. A possible explanation for this finding includes the differences in failure mode as well as pre‐existing PPM in the Epic group [9]. Compared to other surgical aortic bioprostheses, the deterioration of Trifecta prostheses includes an increased rate of cusp tear and subsequent prosthesis regurgitation [26, 27, 28, 29]. Although reduced EOA postprocedure did not translate into statistically worse hemodynamic performance in terms of mean or peak valve gradient in our study, valve‐in‐Epic resulted in mild prosthesis stenosis based on mean gradient and valve opening area, a finding that is in line with other reports on aortic ViV‐TAVR [9, 29, 30]. It is known that PPM is associated with impaired long‐term survival following valve replacement [24]. In our sample, more than half of the patients discharged with at least moderate PPM were not high‐risk based on the STS‐score. For these patients, elective rSAVR may be considered as it could lead to improved hemodynamics and therefore better long‐term clinical outcomes [3, 31, 32].

### 4.1 Limitations

The current study has several limitations. First, we focused on the Trifecta prostheses representing a valve design with externally mounted leaflets and our results are therefore limited to this particular heart valve. Second, sample size was limited which may explain why we could not detect statistical significance for the primary outcome of interest. Third, some hemodynamic measures were incomplete during follow‐up and hence should be interpreted with caution. Forth, relevant CT‐based measurements such as the valve‐to‐aorta or valve‐to‐coronary distances were available in three cases only and could therefore not be studied for this analysis. Finally, screening processes were not documented for patients presenting with Trifecta or Epic valve failure who were not referred for intervention, and our data therefore carries a risk of selection bias.

## 5 Conclusions

In comparison to a standard surgical aortic bioprosthesis without increased risk of CO based on mounting of leaflets, Trifecta aortic bioprostheses with an indication for repeat intervention can be treated safely using ViV‐TAVR in the majority of cases, when techniques to limit the risk of CO are appropriately utilized. One year mortality is acceptable post‐ViV TAVR, but a significant proportion of patients have post‐intervention PPM which may affect longer term outcomes.

## Conflicts of Interest

Dr. Borger declares that his hospital receives speakers' honoraria and/or consulting fees on his behalf from Edwards Lifesciences, Medtronic, Abbott, and Artivion. Dr. Abdel‐Wahab declares that his hospital receives speaker's honoraria and/or consultancy fees on his behalf from Medtronic, Boston Scientific and Edwards Lifesciences. Prof Curzen declares receipt of grants from Boston Scientific, HeartFlow, Beckman Coulter, Haemonetics and honoraria from Abbott, Shockwave & Boston. Dr. Dubois declares that he is transcatheter heart valve proctor for Edwards Lifesciences.

## Supporting information

Supporting information.

## Data Availability

The data that support the findings of this study are available on request from the corresponding author. The data are not publicly available due to privacy or ethical restrictions.
