# Supplementary material for: The International TrifectaTM and EpicTM Valve‐in‐Valve Registry: Insights Into Clinical & Hemodynamic Outcomes
Source: Catheter Cardiovasc Interv. 2025 Mar 27;105(7):1711–8. doi: 10.1002/ccd.31492 (PMC12159376; doi:10.1002/ccd.31492)

**Supplement**

**Table S1. Ethic Approval Registration Numbers of the Participating Centers**

| **Center** | **Registration Number** |
| --- | --- |
| Leipzig | 518/19-ek |
| Dresden | BO-EK-140032022 |
| Jena | Reg.-Nr.: 2022-2577_1-Reg |
| Eindhoven | W22.138  nWMO-2022.075 |
| Leuven | S66583 |
| Southampton | REC reference 22/NS/0122  IRAS project ID 315651 |

**Table S2a. Computed Tomography and Procedural Characteristics of Transcatheter Heart Valves Size 23mm**

|  | Balloon-expandable N=18 | self-expandable N=30 | p-value |
| --- | --- | --- | --- |
| Surgical Valve: |  |  | 0.317 |
| Epic | 3 (16.7%) | 10 (33.3%) |  |
| Trifecta | 15 (83.3%) | 20 (66.7%) |  |
| Time between Interventions (years) | 6.33 [4.69;7.26] | 6.65 [5.05;8.05] | 0.394 |
| Surgical Valve Diameter<23mm | 7 (38.9%) | 16 (53.3%) | 0.502 |
| Labelled Surgical Valve Size: |  |  | 0.180 |
| 19 | 1 (5.56%) | 0 (0.00%) |  |
| 21 | 6 (33.3%) | 16 (53.3%) |  |
| 23 | 11 (61.1%) | 14 (46.7%) |  |
| Annulus Maximal Diameter (mm) | 22.0 [21.0;24.5] | 21.0 [20.0;25.1] | 0.705 |
| Annulus Mean Area (mm^2^) | 343 [304;380] | 302 [284;380] | 0.732 |
| Left Coronary Artery Height (mm) | 8.00 [6.40;11.5] | 8.00 [5.50;10.0] | 0.487 |
| Right Coronary Artery Height (mm) | 11.0 [7.00;13.0] | 10.0 [6.95;14.5] | 0.876 |
| SoV Width (mm) | 31.0 [29.6;33.0] | 31.0 [29.8;33.2] | 0.956 |
| STJ Width (mm) | 26.4 [26.0;29.0] | 28.0 [26.2;29.9] | 0.135 |
| Cerebral Protection | 0 (0.00%) | 15 (50.0%) | 0.001 |
| Predilatation | 7 (41.2%) | 7 (23.3%) | 0.340 |
| Any Coronary Protection Maneuver | 1 (25.0%) | 4 (18.2%) | 1.000 |
| Leaflet Laceration (BASILICA) | 0 (0.00%) | 3 (12.5%) | 1.000 |
| Postdilatation | 1 (5.88%) | 12 (40.0%) | 0.017 |

**Table S2b. Computed Tomography and Procedural Characteristics of Transcatheter Heart Valves Size 26mm**

|  | Balloon-expandable N=12 | self-expandable N=11 | p-value |
| --- | --- | --- | --- |
| Surgical Valve: |  |  | 0.300 |
| Epic | 8 (66.7%) | 4 (36.4%) |  |
| Trifecta | 4 (33.3%) | 7 (63.6%) |  |
| Time between Interventions (years) | 3.45 [1.44;4.84] | 8.30 [4.87;9.86] | 0.027 |
| Surgical Valve Diameter<23mm | 1 (8.33%) | 0 (0.00%) | 1.000 |
| Labelled Surgical Valve Size: |  |  | 0.451 |
| 21 | 1 (8.33%) | 0 (0.00%) |  |
| 23 | 4 (33.3%) | 7 (63.6%) |  |
| 25 | 6 (50.0%) | 3 (27.3%) |  |
| 27 | 1 (8.33%) | 1 (9.09%) |  |
| Annulus Maximal Diameter (mm) | 23.0 [22.5;25.2] | 22.5 [21.5;23.0] | 0.378 |
| Annulus Mean Area (mm^2^) | 405 [401;500] | 353 [310;375] | 0.027 |
| Left Coronary Artery Height (mm) | 11.0 [10.2;11.5] | 11.0 [9.00;12.5] | 0.924 |
| Right Coronary Artery Height (mm) | 11.9 [9.10;15.0] | 9.00 [7.00;14.5] | 0.507 |
| SoV Width (mm) | 35.5 [34.5;36.5] | 33.0 [31.0;40.5] | 0.705 |
| STJ Width (mm) | 39.0 [33.3;41.0] | 32.0 [31.0;35.0] | 0.424 |
| Access: transfemoral | 12 (100%) | 10 (90.9%) | 0.478 |
| Cerebral Protection | 0 (0.00%) | 8 (72.7%) | <0.001 |
| Predilatation | 6 (50.0%) | 2 (18.2%) | 0.193 |
| Any Coronary Protection Maneuver | 1 (12.5%) | 2 (20.0%) | 1.000 |
| Leaflet Laceration (BASILICA) | 0 (0.00%) | 1 (10.0%) | 1.000 |
| Postdilatation | 1 (8.33%) | 7 (63.6%) | 0.009 |

**Table S3. Multivariable Regression**

===============================================================

Dependent variable:

---------------------------

Primary Outcome

------------------------------------------------------------------------------------------------------

Mode of Failure (Regurgitation) -4.1** (-7.3, -0.85)

Type of Prosthesis (Trifecta^TM^) 1.3 (-1.6, 4.1)

STS-Score 0.11 (-0.077, 0.3)

Constant -1.6 (-3.7, 0.52)

------------------------------------------------------------------------------------------------------

Observations 49

Log Likelihood -12.000

Akaike Inf. Crit. 33.000

===============================================================

*p<0.1; **p<0.05; ***p<0.01

STS=Society of Thoracic Surgeons

| **Table S4.** Selected Variables of Cases of Coronary Protection or Coronary Obstruction | | | | | | | | | |
| --- | --- | --- | --- | --- | --- | --- | --- | --- | --- |
| Prosthesis | True Internal Diameter (mm) | Maximum Annulus Diameter (mm) | Annulus Mean Area (mm^2)^ | Height Left Main Coronary Artery (mm) | Height Right Coronary Artery (mm) | Sinus of Valsalve Width (mm) | Sinutubular Junction Width (mm) | Basilica Prcoedure | ViV Prosthesis Type |
| Trifecta | 21 | 21 | 314 | NA | NA | 34 | 26 | no | Balloon-expandable |
| Trifecta | 23 | 23 | 390 | NA | NA | 35 | NA | no | Balloon-expandable |
| Trifecta | 19 | 18 | 246 | 8 | 5 | 30 | 30 | yes | self-expandable |
| Trifecta | 21 | 29 | 572 | 9 | 19 | 36 | 30 | no | self-expandable |
| Trifecta | 19 | 20 | 305 | 5 | 7 | 29 | 28 | yes | self-expandable |
| Trifecta | 21 | 23 | 380 | 8 | 9 | 32 | 33 | no | self-expandable |
| Trifecta | 19 | 20 | 283 | 6 | 5 | 31 | 28 | yes | self-expandable |
| Trifecta | 21 | 18 | 227 | 13 | 16 | 21 | 19 | yes | self-expandable |
| Epic | 19 | NA | NA | NA | NA | NA | NA | no | self-expandable |

| Table S5. By-Case Hemodynamic Outcome (at Discharge), grouped by surgical prosthesis and sorted by True internal Diameter | | | | | | |
| --- | --- | --- | --- | --- | --- | --- |
| True Internal Diameter (mm) | **ViV Prosthesis** | **ViV Size (mm)** | **LV-EF (%)** | **PVL > trace** | **Mean Gradient (mmHg)** | **Residual Gradient** |
| Epic | | | | | | |
| 17 | Balloon-expandable | 26 | NA | no | NA | NA |
| 17 | self-expandable | 23 | 34 | no | 6.0 | no |
| 17 | self-expandable | 23 | 65 | yes | 19.2 | yes |
| 19 | Balloon-expandable | 23 | 35 | no | 15.0 | no |
| 19 | self-expandable | 23 | 55 | no | 29.0 | yes |
| 19 | self-expandable | 23 | 47 | no | 14.0 | no |
| 19 | Balloon-expandable | 26 | NA | no | NA | NA |
| 19 | Balloon-expandable | 26 | 32 | no | 20.0 | yes |
| 19 | Balloon-expandable | 26 | 44 | yes | 14.0 | no |
| 19 | Balloon-expandable | 26 | 65 | no | 29.0 | yes |
| 19 | Balloon-expandable | 23 | 42 | no | 21.0 | yes |
| 19 | Balloon-expandable | 23 | 45 | no | 20.0 | yes |
| 19 | self-expandable | 23 | 53 | yes | 33.4 | yes |
| 19 | self-expandable | 23 | 30 | yes | 8.0 | no |
| 19 | Balloon-expandable | 20 | 32 | no | 42.0 | yes |
| 19 | self-expandable | 23 | 45 | yes | 21.0 | yes |
| 19 | self-expandable | 26 | 67 | no | 10.0 | no |
| 19 | self-expandable | 23 | 55 | no | NA | NA |
| 19 | self-expandable | 26 | 42 | no | NA | NA |
| 19 | self-expandable | 23 | NA | no | 13.0 | no |
| 19 | self-expandable | 23 | 42 | no | 19.0 | no |
| 21 | Balloon-expandable | 26 | 64 | yes | 32.0 | yes |
| 21 | Balloon-expandable | 26 | 60 | no | 11.0 | no |
| 21 | Balloon-expandable | 29 | 35 | no | 8.0 | no |
| 21 | self-expandable | 26 | 50 | yes | 14.0 | no |
| 21 | self-expandable | 26 | NA | no | 9.0 | no |
| 23 | Balloon-expandable | 26 | 60 | yes | 17.9 | no |
| Trifecta | | | | | | |
| 17 | Balloon-expandable | 23 | 55 | no | 37.0 | yes |
| 19 | self-expandable | 23 | NA | no | NA | NA |
| 19 | self-expandable | 23 | 21 | no | 9.0 | no |
| 19 | self-expandable | 23 | 49 | no | 6.0 | no |
| 19 | self-expandable | 23 | 82 | no | 19.0 | no |
| 19 | self-expandable | 23 | 68 | no | 9.0 | no |
| 19 | self-expandable | 23 | 55 | no | 8.0 | no |
| 19 | self-expandable | 23 | 60 | yes | 6.0 | no |
| 19 | self-expandable | 23 | 43 | no | 8.2 | no |
| 19 | self-expandable | 23 | 46 | no | 13.0 | no |
| 19 | self-expandable | 23 | 41 | no | 21.0 | yes |
| 19 | self-expandable | 23 | 66 | no | 23.0 | yes |
| 19 | self-expandable | 23 | 66 | no | 27.0 | yes |
| 19 | Balloon-expandable | 23 | NA | no | NA | NA |
| 19 | self-expandable | 23 | 50 | yes | 12.0 | no |
| 19 | Balloon-expandable | 23 | 50 | no | 17.5 | no |
| 19 | Balloon-expandable | 23 | 50 | no | 21.0 | yes |
| 19 | Balloon-expandable | 23 | 20 | no | 26.2 | yes |
| 19 | Balloon-expandable | 23 | 55 | no | 16.0 | no |
| 19 | Balloon-expandable | 23 | 60 | yes | 17.2 | no |
| 19 | self-expandable | 23 | NA | no | 3.0 | no |
| 21 | self-expandable | 29 | 65 | yes | 46.8 | yes |
| 21 | Balloon-expandable | 23 | 54 | no | 17.4 | no |
| 21 | self-expandable | 23 | 60 | yes | 16.0 | no |
| 21 | self-expandable | 23 | 67 | no | 13.0 | no |
| 21 | self-expandable | 26 | 52 | no | 12.0 | no |
| 21 | Balloon-expandable | 23 | 60 | no | 8.4 | no |
| 21 | Balloon-expandable | 23 | NA | no | 31.0 | yes |
| 21 | Balloon-expandable | 23 | 50 | no | 18.0 | no |
| 21 | self-expandable | 23 | 43 | no | 22.0 | yes |
| 21 | self-expandable | 23 | 49 | yes | 12.0 | no |
| 21 | self-expandable | 26 | 46 | no | 7.0 | no |
| 21 | self-expandable | 26 | 63 | no | 12.0 | no |
| 21 | self-expandable | 26 | 54 | no | 12.0 | no |
| 21 | self-expandable | 26 | 23 | no | 16.0 | no |
| 21 | Balloon-expandable | 23 | NA | no | NA | NA |
| 21 | self-expandable | 23 | 51 | no | NA | NA |
| 21 | Balloon-expandable | 23 | 45 | no | 31.0 | yes |
| 21 | Balloon-expandable | 23 | 50 | no | 15.9 | no |
| 21 | Balloon-expandable | 23 | 50 | no | 26.5 | yes |
| 21 | self-expandable | 23 | 77 | no | NA | NA |
| 23 | Balloon-expandable | 26 | 60 | no | 18.0 | no |
| 23 | Balloon-expandable | 26 | 55 | no | 13.6 | no |
| 23 | self-expandable | 26 | 49 | no | 5.6 | no |
| 23 | self-expandable | 29 | 22 | yes | 6.3 | no |
| 23 | self-expandable | 29 | 51 | yes | 13.0 | no |
| 23 | Balloon-expandable | 26 | 55 | no | 14.0 | no |
| 23 | Balloon-expandable | 26 | 20 | yes | 16.8 | no |
| 25 | self-expandable | 26 | NA | yes | NA | NA |

**Figure S1.** Plot depicting mean AV gradient at discharge (y-axis) by true internal diameter (x-axis), for Epic (blue) and Trifecta (orange) bioprostheses. Shape indicates moderate or severe patient prosthesis mismatch (triangle)


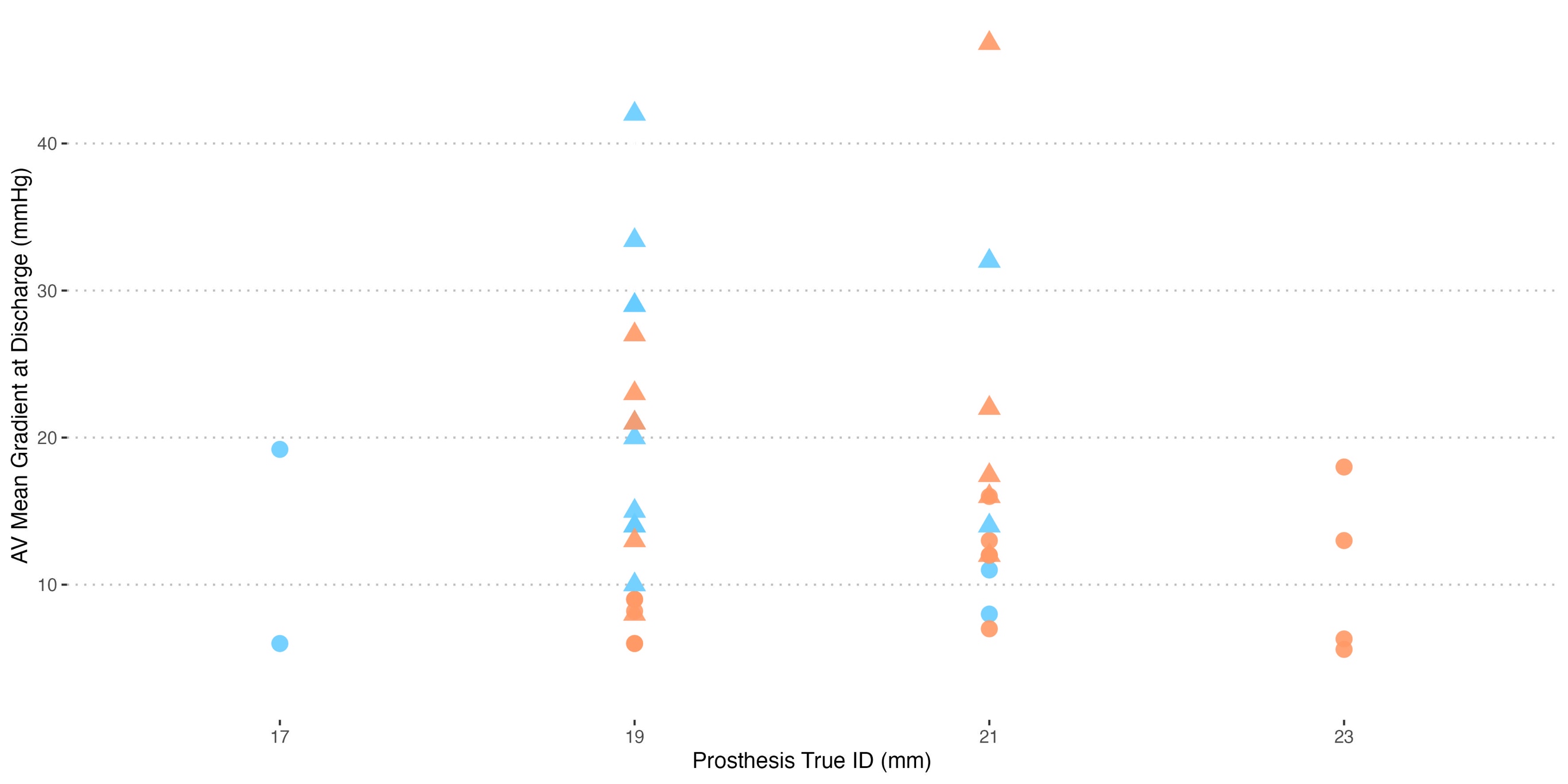

Supplement: Supplementary file 1 — Supporting information. [file CCD-105-1711-s001.docx]
